# PMC42, a breast progenitor cancer cell line, has normal-like mRNA and microRNA transcriptomes

**DOI:** 10.1186/bcr2109

**Published:** 2008-06-27

**Authors:** Anna Git, Inmaculada Spiteri, Cherie Blenkiron, Mark J Dunning, Jessica CM Pole, Suet-Feung Chin, Yanzhong Wang, James Smith, Frederick J Livesey, Carlos Caldas

**Affiliations:** 1 Breast Cancer Functional Genomics Laboratory, Cancer Research UK Cambridge Research Institute and Department of Oncology, University of Cambridge, Li Ka Shing Centre, Robinson Way, Cambridge CB2 0RE, UK; 2 Department of Pathology, Hutchison-MRC Research Centre, University of Cambridge, Hills Road, Cambridge CB2 2XZ, UK; 3 Current address: Robertson Centre for Biostatistics, Boyd Orr Building, University of Glasgow, Glasgow G12 8QQ, UK; 4 Gurdon Institute and Department of Biochemistry, University of Cambridge, Tennis Court Road, Cambridge CB2 1QN, UK

## Abstract

**Introduction:**

The use of cultured cell lines as model systems for normal tissue is limited by the molecular alterations accompanying the immortalisation process, including changes in the mRNA and microRNA (miRNA) repertoire. Therefore, identification of cell lines with normal-like expression profiles is of paramount importance in studies of normal gene regulation.

**Methods:**

The mRNA and miRNA expression profiles of several breast cell lines of cancerous or normal origin were measured using printed slide arrays, Luminex bead arrays, and real-time reverse transcription-polymerase chain reaction.

**Results:**

We demonstrate that the mRNA expression profiles of two breast cell lines are similar to that of normal breast tissue: HB4a, immortalised normal breast epithelium, and PMC42, a breast cancer cell line that retains progenitor pluripotency allowing in-culture differentiation to both secretory and myoepithelial fates. In contrast, only PMC42 exhibits a normal-like miRNA expression profile. We identified a group of miRNAs that are highly expressed in normal breast tissue and PMC42 but are lost in all other cancerous and normal-origin breast cell lines and observed a similar loss in immortalised lymphoblastoid cell lines compared with healthy uncultured B cells. Moreover, like tumour suppressor genes, these miRNAs are lost in a variety of tumours. We show that the mechanism leading to the loss of these miRNAs in breast cancer cell lines has genomic, transcriptional, and post-transcriptional components.

**Conclusion:**

We propose that, despite its neoplastic origin, PMC42 is an excellent molecular model for normal breast epithelium, providing a unique tool to study breast differentiation and the function of key miRNAs that are typically lost in cancer.

## Introduction

Despite its many shortcomings, continuous cell culture remains the most common model system for investigating molecular mechanisms of normal differentiation, disease, and neoplastic transformation (reviewed in [1]). This is thanks mostly to ease of use, homogeneity, availability in large quantities, and sustainability over prolonged periods of cell culture. Cell lines typically are established either from tumours (primary or metastatic) or from immortalised normal cells often used as experimental models of healthy tissue. However, the normal physiology of many cell types depends on their native neighbouring cell types and extracellular matrix (ECM), exemplified in the context of the breast by the dependence of luminal (epithelial/secretory) cells on their basal (myoepithelial) neighbours and surrounding ECM components for maintenance of polarity and differentiation [2,3]. Thus, isolation of individual cells for their propagation in culture often leads to partial loss of normal phenotype. Moreover, both one-off major changes incurred on the cell by the immortalisation process (be it natural or induced) and gradual drifts in karyotype, growth, and differentiation characteristics affect all cell lines and do not spare those with normal ancestry. Taken together, these limitations raise the question of whether immortalised normal cells are necessarily the optimal choice to represent healthy tissue, or in other words, what underlies the 'normality' of the healthy tissue and whether these features are maintained in culture. The matter is by no means a new one. A review published 17 years ago by Mina Bissell [4] summarised debates that at the time surrounded morphological, metabolic, and protein expression aspects of cell lines and their validity as representatives of normal tissue. Unfortunately, it appears that the progress of cell and molecular biology has done little to relieve the controversies since. Despite the ability to do genome-wide profiling and the ever-expanding list of available 'tumour markers', we are still unable to define what constitutes a good model system for normal gene expression.

PMC42 is a breast carcinoma cell line established from a pleural effusion of a woman with metastatic breast cancer [5-7]. Despite bearing some hallmark features of other cancer cell lines (namely, loss of contact inhibition and a highly rearranged karyotype), PMC42 is remarkably similar to normal breast epithelial tissue, suggesting that unlike most common breast cancer cell lines stemming from tumours of luminal or myoepithelial/basal cells, PMC42 is a rare cell line originating from a breast stem cell retaining much of the breast's native phenotype. First, PMC42 exhibited stable heterogeneity of at least eight morphologically distinct cell types arising from in-culture differentiation of both epithelial and myoepithelial progeny all the way to terminal differentiation and cell death. Second, the cells were shown to respond to hormonal stimuli in a manner similar to breast epithelial tissue, such as the appearance of lipid droplets upon treatment with prolactin or stimulation of growth by hydrocortisone, in marked contrast to other breast tumour cell lines inhibited by this glucocorticoid [7]. Moreover, the cord-like subpopulation of PMC42 not only responded to epithelial growth factor (EGF) stimulus but also could attach to collagen and laminin matrices. EGF stimulation of monolayer PMC42 induced morphological, biochemical, and phenotypical changes reminiscent of epithelio-mesenchymal transition *in vivo *[8]. Lastly, it was demonstrated that, by exposing PMC42 grown on Matrigel-coated plates to a combination of hormonal stimuli, the cells could be driven to form hollow organoids expressing β-casein [9], thus fulfilling two additional criteria commonly used for normal breast epithelial differentiation.

Understanding stem cell self-renewal and cellular differentiation is one of the current challenges in biomedical research. The current paradigm considers both intrinsic (for example, transcription factors) and extrinsic (for example, signalling pathways) contributions to the balance between self-renewal and differentiation (reviewed in [10]). In recent years, it has become increasingly clear that post-transcriptional regulation of gene expression by microRNAs (miRNAs) is a major intrinsic component in numerous systems, including neuronal differentiation, the development of skeletal and cardiac muscle, and the formation of the haematopoietic system (reviewed in [11-13], respectively). Moreover, since their discovery in 1993, miRNAs have rapidly secured an undisputed status as key players in malignancy [14,15]. We therefore undertook to examine the gene expression profiles underlying the normal-like phenotype of PMC42 in contrast to other commonly used breast cell lines (normal and cancer-derived).

## Materials and methods

### General comments

All oligonucleotide sequences are listed 5'-3' (uppercase = DNA, lowercase = RNA, p = phosphate). Annotation of miRNAs varied slightly between platforms. Thus, where considered helpful, annotation was manually unified.

### Cell culture and isolation of total RNA and small-RNA fractions

The following adherent cell lines were used for the core findings of this work: oestrogen receptor/progesterone receptor (ER/PR)-positive normal immortalised breast epithelium HB4a (from the originator) [16], ER/PR-positive basal/progenitor breast cancer PMC42 (from Michael O'Hare) [5], ER/PR-negative basal breast cancer BT-20 (from Michael O'Hare) [17], ER/PR-negative luminal breast cancer HCC1419 (from American Type Culture Collection [ATCC], Manassas, VA, USA) [18], ER/PR-positive breast cancer MCF7 (from ATCC) [19], ER/PR-positive MDA-MB-361 (from ATCC) [20], and ER/PR-negative MT-3 (from the originator [21], originally isolated as a breast cancer line, later determined to be LS-174T, a colon cell line [22]). All of the above cell lines have been previously karyotyped [23,24].

For validation of miRNA expression, we have used the immortalised normal epithelial breast cell lines HMT-3522 [25] and MCF-10A [26] (from ATCC) as well as the following breast cancer cell lines (from Paul Edwards) ([24] and references therein): T47-D, SUM190, OCUBF, MDA-MB-468, SK-BR-3, SUM-52, SK-BR-7, HCC1187, CAMA-1, MDA-MB-231, and HCC1937.

The ER and PR status of the cell lines was obtained from the references above and from publicly available databases [27-29]. Where available, the luminal/basal nature of the cell lines was obtained from [30] and verified by quantitative reverse transcription-polymerase chain reaction (qRT-PCR) of selected genes (data not shown). For ease of annotation, hyphens have been omitted from the cell line names throughout the rest of the manuscript.

Cells were cultured according to suppliers' recommendations. RNA was harvested from subconfluent cultures (estimated 85% density) that were re-fed with fresh medium 24 hours prior to harvesting. In brief, cultures were washed once with cold phosphate-buffered saline (PBS). Upon complete removal of the PBS, cells were lysed directly in 8 mL of TRI reagent (Sigma-Aldrich, St. Louis, MO, USA) and processed according to manufacturer's protocol with the following modification: precipitation of RNA out of the aqueous phase was performed overnight at -20°C in the presence of 1.7 volumes of 2:1 ethanol/isopropanol and additional 0.3 M NaOAc (pH 5.5). Small RNAs were further purified using the miRVANA kit (Ambion, Austin, TX, USA). The yield and quality of the small RNAs were monitored by electrophoresis of approximately 1 μg of total RNA and the corresponding proportion of the small-RNA fraction through a 15% acrylamide/urea vertical gel and subsequent staining with 1:10,000 SYBR Gold (Molecular Probes, now part of Invitrogen Corporation, Carlsbad, CA, USA). Small-RNA fractions were extracted with a near 100% efficiency, comprised 7% to 17% of the total RNA, and contained predominantly tRNAs and small rRNA. No miRNA-sized RNA was ever visible in miRVANA fractions from cell lines (<0.5 ng). Total RNA from individual normal breast (from Ambion and Stratagene [La Jolla, CA, USA], termed throughout the paper as NormalA and NormalS, respectively) was similarly processed for small-RNA fractions.

### mRNA profiling on beadchip arrays

Total RNA was labelled using the Illumina TotalPrep RNA Amplification kit (Ambion) in accordance with the manufacturer's instructions. Briefly, 200 ng of total RNA was reversed-transcribed in a 20-μL reaction for 2 hours at 42°C. Second-strand cDNA synthesis was performed at 16°C for 2 hours, and the resulting double-stranded cDNA was purified and washed using filter columns provided with the kit. Finally, the cDNA was transcribed *in vitro *at 37°C for 14 hours using T7 RNA polymerase and a biotin-NTP mix. Upon completion, the reaction was diluted with 75 μL of nuclease-free water and the cRNA was purified and washed using filter columns to retrieve a 100-μL eluate.

Biotin-labelled cRNA (1.5 μg) in 20 μL was used for each hybridisation on Sentrix Human-6 BeadChips (Illumina, San Diego, CA, USA) in accordance with manufacturer's protocol. In brief, samples were mixed with 40-μL hybridisation mix (3:2 hybridisation buffer/deionised formamide) and preheated at 65°C for 5 minutes. The 60-μL assay sample was dispensed onto the array immediately after cooling to room temperature and incubated for 18 hours at 55°C. After the overnight incubation, the slides were washed at 55°C followed by three room temperature washes. Finally, the slides were blocked and the cRNA was detected by a 10-minute incubation with streptavidin-Cy3. After an additional wash at room temperature, the slides were spin-dried and scanned using the default settings of the Illumina BeadArray reader (Illumina).

Illumina's BeadStudio software (version 1.5.1.3) was used to process raw array data according to [31] and to output a single summarised value for each of the 48,000 genes on each array. These non-normalised data were then read into the Bioconductor bead array [31] software for quality control and normalisation. Quality control checks showed low variability between replicate arrays and no evidence of experimental bias between arrays. The data were then normalised by scaling to the global median or by quantile normalisation and are available on ArrayExpress under accession number E-TABM-194 (temporary username: Reviewer_E-TABM-194, password: 1166801733027). An empirical Bayes analysis of variance approach [32] was then used on the normalised data to separate genes that were differentially expressed between samples from genes with equal expression level across all samples. The result was a list of B-statistics for each gene under the two normalisation approaches, with a larger statistic indicating greater evidence for differential expression. Genes with B-statistics of greater than 10 for either normalisation were then selected for further analysis. Gene ontology analysis was performed using the web-based FatiGo software [33,34].

**Figure 1:**
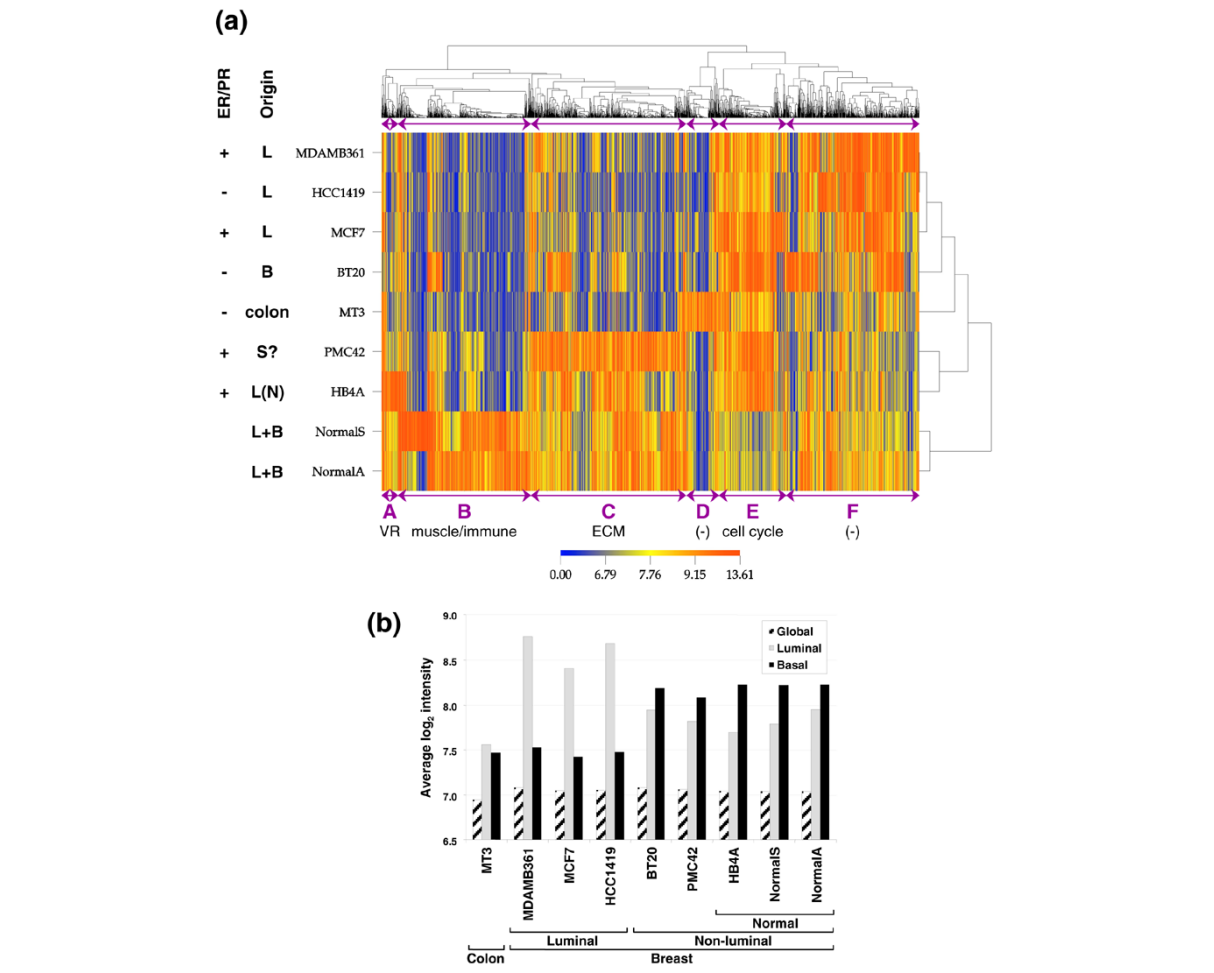
Analysis of mRNA gene expression in breast cell lines and normal tissue. **(a) **Analysis of differentially expressed mRNAs. Clustering heatmap for genes with analysis of variance B values of greater than 10 after either a median or a quantile normalisation. Cell type origin (B, basal; L, luminal; N, normal; S?, possible stem cell) and status of oestrogen receptor/progesterone receptor (ER/PR) are indicated on the left. mRNA clusters are indicated by purple letters (below, with their key enriched process) and purple arrows (above and below). ECM, extracellular matrix; VR, viral response. **(b) **Analysis of luminal/basal mRNA markers. The average log_2 _intensity of expression of 252 luminal and 360 basal annotated non-redundant markers is shown.

The genes used for luminal/basal analysis in Figure 1b were based on the recently reported luminal/basal gene set signature in breast cell lines (1,233 genes [30]), out of which we selected 612 (252 luminal and 360 basal) genes for which a unique gene symbol annotation was available in the original study and for which only one probe was present in our beadchip array.

### MicroRNA profiling on slide arrays

Individual small-RNA fractions or a pool containing equal quantities of small-RNA fractions from the six cancer cell lines was labelled with a Genisphere Array 900 miRNA RT kit (MIRT180; Genisphere, Hatfield, PA, USA) in accordance with manufacturer's instructions. Briefly, 500 ng of small-RNA fraction was polyA-tailed for 15 minutes at 37°C using poly(A) polymerase (PAP). The tailed samples were then reverse-transcribed using Superscript II (Invitrogen Corporation) for 1 hour at 42°C and one of two capture primers (Cy3-capture primer TTCTCGTGTTCCGTTTGTACTCTAAGGTGGA-T_17_; Cy5-capture primer ATTGCCTTGTAAGCGATGTGATTCTATTGGA-T_17_) spanning a capture sequence later to be detected by complementary oligonucleotides tagged with approximately 850 fluorophor moieties each, included in the kit. Upon completion, the two cDNAs (primed with either Cy3-capture or Cy5-capture) were combined and concentrated with Microcon YM-50 columns (Millipore Corporation, Billerica, MA, USA) to a final volume of 25 μL.

The profiling was performed on homemade arrays bearing 171 miRNA, 4 tRNA, and 3 snRNA amine-modified probes synthesised in sense orientation by QIAGEN-Operon and spotted in 32 or 64 replicates on amine-reactive Codelink slides (GE Healthcare, Little Chalfont, Buckinghamshire, UK). Prior to the hybridisations, the slides were blocked in a 1% wt/vol ammonium hydroxide solution for 5 minutes with gentle shaking, rinsed in water, and spin-dried. Labelled samples were mixed with 25 μL of MIRT180 hybridisation buffer and incubated at 80°C for 10 minutes and then at 37°C until loading onto a pre-warmed array. After an overnight incubation at 37°C in a dark humidified chamber, the slides were washed for 15 minutes at 42°C in pre-warmed 2× Saline Sodium Citrate (SSC), 0.2% SDS buffer, 15 minutes in 2 × SSC at room temperature, and 15 minutes in 0.2 × SSC at room temperature and, finally, spin-dried. Cy3 and Cy5 detection reagents were mixed with the hybridisation buffer in a 25-μL reaction mix and incubated at 80°C for 10 minutes and then at 64°C until loading onto a pre-warmed array. After 4 hours of incubation at 64°C in a dark humidified chamber, the slides were washed as before and scanned on a DNA microarray scanner (G2565AA Microarray Scanner System; Agilent Technologies, Santa Clara, CA, USA). Each sample was hybridised to four slides (two duplicates for each Cy3/Cy5 dye swap), except HB4a, for which the dye swap was performed only once.

Microarray images were analysed by using GenePix Pro software version 4.1 (Molecular Devices Corporation, Sunnyvale, CA, USA), and further computational analysis of the miRNA expression data was performed using Gene Expression Pattern Analysis Suite version 2.0 [35]. Data were normalised using the DNAMD (Diagnosis and Normalization for MicroArray Data) tool [36]. User-defined settings were (a) GenePix negative-flagged spots for the print-tip loess, which were included for intra-array normalisation but excluded from any further analysis, and (b) a background-half correction method. Inconsistent replicates (average distance to the median of greater than 0.5) were removed after the normalisation step using the Preprocessing tool [37]. Data are available on ArrayExpress under accession number E-UCON-2 (temporary username: Reviewer_E-UCON-2, password: 1169041712146). Only data from miRNAs with signal intensity (A values) greater than 6 were considered reliable and taken for further analysis. To account for variable probe-specific dye bias, final M values were calculated as the difference between the average log_2 _(ratio) values from Cy3 and Cy5 dye swaps. Differentially expressed miRNAs were defined as the genes for which at least one value was up- or down-expressed compared with the pool with log_2 _greater than 2.5. M values of differentially expressed or all miRNAs were clustered by CIMminer using correlation distances with 'cluster method: average' [38,39].

### MicroRNA profiling on Luminex bead arrays

Following the method described in [40], miRNAs were extracted from 5 μg of total RNA using denaturing PAGE. Samples were spiked with three synthetic pre-labelling control RNAs (3 fmol per sample; pCAGUCAGUCAGUCAGUCAGUCAG, pGACCUCCAUGUAAACGUACAA, and pUUGCAGAUAACUGGUACAAG; Dharmacon, Chicago, IL, USA) to monitor target preparation efficiency. After purification of the small 18- to 26-base pair RNAs, they were ligated at the 3' and 5' end to adapters using T4 RNA ligase (3' adapter – puuuAACCGCGAATTCCAGT; 5' adapter – ACGGAATTCCTCACTaaa). Bi-ligated products were reverse-transcribed using an adaptor-specific primer (M37 – TACTGGAATTCGCGGTTA) and then amplified and biotin-labelled by PCR (M37 and M33 – biotin-CAACGGAATTCCTCACTAAA) for 18 cycles of 95°C for 30 seconds, 50°C for 30 seconds, and 72°C for 40 seconds. PCR products were precipitated without glycogen and redissolved in 66 μL 1× TE buffer containing 1 μL of three biotinylated post-labelling controls (100 fmol each, FVR506, PTG20210, and MRC677).

Four bead sets were constructed containing 90 different coded beads each. Altogether, they were used to profile 310 miRNAs, including all human miRNAs known at the time, six labelling controls and six bead repeats common to all bead sets to aid subsequent normalisation. Each labelled sample was hybridised to the four bead sets to generate a complete miRNA profile. Oligos were 5'-amino modified with a 6-carbon linker and conjugated to carboxylated xMAP beads (Luminex Corporation, Austin, TX, USA) in 96-well formats following the standard protocol of the manufacturer. To generate bead set pools, 3 μL of each oligo-bead conjugate was mixed into 1-mL 1.5× tetramethylammonium chloride (TMAC) buffer (Luminex Corporation). Samples were hybridised in a 96-well format alongside background controls, two water blanks, and at least three bead blanks containing water instead of the labelled sample for use as a background control. Repeat samples were included across bead sets and sample plates. Hybridisation was carried out overnight at 50°C with 33 μL of the bead pool and 15 μL of labelled sample. Prior to reading on the Luminex, unbound sample was removed from beads by washing with 1 × TE (Tris + EDTA [ethylenediaminetetraacetic acid]) and resuspending in 1 × TMAC buffer. Streptavidin-phycoerythrin (premium grade; Invitrogen Corporation) was added to the beads (1:100 dilution) and incubated for 10 minutes at 50°C to bind to biotin moieties on the cDNA. Samples were processed on a Luminex 100 machine, and median fluorescence intensity values were measured. Data are presented in this paper without any further manipulation.

### Real-time reverse transcription-polymerase chain reaction analysis of microRNAs

Real-time RT-PCR (qRT-PCR) analysis of miRNAs was performed essentially as previously described [41]. The small-RNA fraction (500 ng) was polyadenylated by PAP (Ambion) at 37°C for 1 hour in a 6-μL reaction. After phenol-chloroform extraction and ethanol precipitation, the polyadenylated small RNAs were reversed-transcribed according to the manufacturer's protocols using 200 U SuperScript-II Reverse Transcriptase (Invitrogen Corporation) and 0.5-μg poly(T) adapter (GCGAGCACAGAATTAATACGACTCACTATAGGTTTTTTTTTTTTVN). The small cDNAs were then treated with RNAse H at 37°C for 1 hour and finally diluted 240-fold. Negative controls were included in both PAP and RT reactions. Moreover, two additional RT reactions were performed using half and double the input of polyadenylated small RNAs to ensure the linearity of the RT step. The melting temperature for each miRNA was experimentally determined from the thermal dissociation curves using ABI Prism 7900 HT (Applied Biosystems, Foster City, CA, USA).

Finally, the real-time PCR step was carried out in triplicate at the optimal temperature and typically contained 5 pmol of the gene-specific primer, 5 pmol of the common antisense oligonucleotide (GCGAGCACAGAATTAATACGACTC), 10 μL SYBR Green PCR Master Mix (Applied Biosystems), and 6 μL of the diluted cDNAs in a total volume of 20 μL. Each set of PCRs included a dilution series of a reference cDNA to be used for quantitation. The sequences of forward primers for mature miRNAs were identical to the complete miRNA sequence listed in the miRBASE database. Additional forward primers were 5S rRNA ACCGGGTGCTGTAGGCT, Pre-mir-127 GGTCGGAAGTCTCATC, Pre-mir-126 GCTGGCGACGGGACAT, Pre-mir-143 CGCCCTGTCTCCCAGCCT, Pre-mir-145 GATGCTAAGATGGGGATTC, Pre-mir-155 TGCCTCCAACTGACTCCTAC, Pre-mir-199a-1 GTTCAGGAGGCTCTCAATGTG, Pre-mir-199a-2 GACTGGGCAAGGGAGAGCA, and Pre-mir-214 GCAGAACATCCGCTCACC.

The following quality control was applied before data sets were accepted: (a) both PAP and RT negative controls gave negligible values or displayed dissociation curves that were significantly different from those in experimental samples, (b) the real-time PCR values of samples containing RT reactions programmed with different RNA inputs showed a dose response or at the very least no reverse response, and (c) the linear fit of the quantitative PCR values of the dilution series was greater than 90%. As the units describing miRNA quantities were arbitrary, data were scaled within each gene analysed so that the highest-expressing sample was set to 1 and, unless otherwise indicated, was not further normalised.

### Array comparative genomic hybridisation on bacterial artificial chromosome arrays

Comparative genomic hybridisation (CGH) on bacterial artificial chromosome (BAC) arrays was performed as described [42,43], essentially according to the methods of [44] using an in-house array [45] comprising DNA amplified from BACs 10 Mb or less apart across the whole genome spotted in triplicate onto amine-binding slides (CodeLink Activated Slides; Amersham Biosciences, now part of GE Healthcare) using a MicroGrid II arrayer (BioRobotics, Boston, MA, USA).

Labelling and hybridisation protocols were as described in [44] with slight modifications. Pre-hybridisation and hybridisation volumes were scaled down for an array surface of 2 × 2 cm delimited by an adhesive plastic frame and performed in humid hybridisation chambers (Camlab Limited, Cambridge, UK). Slides were washed in PBS/0.05% Tween-20 for 10 minutes at room temperature before and after a main wash in 50% formamide/0.5 × SSC for 30 minutes at 42°C.

Arrays were scanned using an Axon Genepix 4100 and the data were acquired using Genepix4.1 software (Molecular Devices Corporation) to perform segmentation and calculate intensities after background subtraction. Spots with intensity below twice the median intensity of the Drosophila clones were rejected. Test/reference ratios were then calculated and normalised to the median ratio of the autosomal chromosome clones. Spots with ratios more than 10% different from the median of the triplicate were rejected. If a minimum of two spots of the triplicate were accepted, the mean of the log_2 _ratios was calculated. For ease of annotation, data points differing from the normal reference sample with a log_2 _ratio of greater than 0.2 or less than -0.2 were considered gains or losses, respectively.

### Array comparative genomic hybridisation on oligonucleotide arrays

DNA was extracted from cell lines using a standard SDS/Proteinase K method, quantified with a NanoDrop ND-1000 spectrophotometer (NanoDrop Technologies, Wilmington, DE, USA), and labelled using the BioPrime DNA labelling system (Invitrogen Corporation). Labelled DNA was hybridised as described previously [46] to oligonucleotide arrays containing 60-mer oligonucleotides representing 28,830 unique genes as designed by Compugen (San Jose, CA, USA). The array construction has been described previously [46], and the complete data and analysis have been reported elsewhere [47].

Fluorescence ratios of scanned images of arrays were obtained using Bluefuse version 3.2 (BlueGnome, Cambridge, UK). After mapping to Human Mar. 2006 assembly (hg18), 27,801 unique positions were identified. The log_2 _ratios were then normalised to their mode values by using the R/Bioconductor package l*imma *[48]. For ease of annotation, data points differing from the normal reference sample with a log_2 _ratio of greater than 0.5 or less than -0.5 were considered gains or losses, respectively.

### Consent and licenses

All research was carried out in compliance with ethics guidelines and regulations. Human B cells were purified from waste products of blood donations with approval from the Addenbrookes Hospital Local Research Ethics Committee.

## Results

### mRNA expression profiling and gene ontology analysis

First, we compared the mRNA expression profile of PMC42 with a panel of commonly used breast cancer cell lines as well as immortalised normal breast epithelium. One thousand nine hundred forty-three genes with significantly differential expression were used for unsupervised clustering (Figure 1a). We observed that the resulting tree was largely in agreement with the founding cell type and not the ER/PR status. The primary separation was between normal breast tissue (NormalA and NormalS) and cell lines. This most likely represents the mixed population of cells (for example, epithelial, stromal, and inflammatory) contained within the Normal samples (see below). Importantly, the readings of the epithelial genes were of similar intensities in the Normal and cell line samples, showing that the mixed nature of the Normal sample does not preclude it as a reference. Amongst the cell lines, PMC42 clustered with HB4a. The other four breast cancer cell lines were clustered together, with the luminal lines on one branch and BT20, a basal breast cancer line, on another (classification based on [30] and references therein). HCC1419 and MDAMB361 were particularly similar despite their opposite ER/PR status. MT3, a colon cell line, clustered with the non-PMC42 breast cancer cell lines.

We then examined the gene ontology terms enriched in the mRNAs clusters underscoring the relationships between the samples (A-F in Figure 1a). Cluster E spanned 244 genes overexpressed in all cell lines compared with normal tissue and contained a high proportion of genes related to cell division and DNA repair (approximately 35% of the genes; *P *values down to 2.94 × 10^-18^), reflecting the high rate of cell cycle of immortalised cell lines. Cluster B (underexpressed in all cell lines compared with normal tissue) contained 479 genes with enrichment in muscle-related functions (approximately 25% of the genes; *P *values down to 5.10 × 10^-4^) and genes involved in immune or inflammatory response (approximately 20% of the genes; *P *values down to 4.37 × 10^-5^). Both of these gene classes probably originate from the non-epithelial components of breast tissue which are absent in the cell lines. Genes related to viral response were also noted in cluster A, spanning 61 genes that were high in both normal tissue and the cell line derivative of normal breast epithelium (HB4a) compared with cancer cell lines (*P *values down to 7.35 × 10^-5^). Interestingly, HB4a also expresses high levels of the interferon-response gene cluster [49].

Of particular interest was cluster C, spanning 462 genes that were high in normal tissue, immortalised breast epithelium, and PMC42. This cluster was rich in genes related to ECM, including basement membrane (BM) (17% of the genes; *P *values down to 5.48 × 10^-4^). Jongeneel and colleagues [50] have reported that HB4a abundantly expressed components of the ECM. Recent views attributing cell-cell and cell-ECM interactions with a major role in the suppression of breast neoplasia ([3] and references therein) propose a significant biological context for this gene cluster. Moreover, it has been demonstrated that micro-environmental signals can override genetic infidelity [51,52]. When clustering was performed excluding HB4a, the mRNA cluster overexpressed in normal and PMC42 samples also contained genes related to fat metabolism, reflecting the milk-producing nature of these cells (data not shown). No significantly enriched gene ontology terms were found in cluster F (481 genes that were highly expressed in all cell lines except PMC42 and HB4a) or D (116 genes highly expressed in the colon line MT3). A full listing of ontology terms and the corresponding genes identified by FatiGO is available as Supplementary file 1.

We then tested whether the segregation of PMC42 from the other breast cancer cell lines merely reflects a difference in their luminal/basal nature. Recent attempts to pinpoint the gene expression signature underlying the classification of breast samples [for example, [30,49,53,54]] revealed gene sets that primarily divide breast samples into two main categories, the luminal and the non-luminal, each of which can be subsequently subdivided into finer groups. We therefore examined the expression of several such gene sets in our samples. Figure 1b shows the average log_2 _expression values for luminal and basal markers, as well as overall intensities of gene expression, of a classifying gene set identified in breast cell lines [30]. While the overall signal intensity on the arrays was similar, the intensities of the luminal/basal markers offered a clear separation of breast samples into luminal and non-luminal groups. The former, showing a high expression of luminal markers and a low expression of basal markers, contained cell lines arising from luminal progenitors (namely, MDAMB361, MCF7, and HCC1419) ([30] and references therein). The non-luminal group, showing lower levels of luminal markers and high levels of basal markers, encompassed the previously clustered normal breast tissue, HB4a and PMC42, but also BT20 (a known basal cell line, confirmed by cytokeratin 5/6 immunostaining; data not shown). Therefore, the overall clustering of PMC42 and HB4a with normal breast tissue was not due solely to the basal nature of these cell lines since BT20, a known basal cell line, unequivocally clustered with the luminal cell lines (Figure 1a). MT3, a colorectal cancer cell line, showed low levels of both types of markers.

Comparison of the mRNA profiles to the gene expression classifiers described for breast tumours [54] consistently identified the normal breast samples as Class-Normal, MDAMB361 and HCC1419 as Luminal type A, and MCF7 as Luminal type B. BT20, PMC42, and HB4a clustered with the Basal and Her2 classifiers but their precise hierarchy varied depending on the algorithm employed (data not shown). PMC42 and HB4a also expressed many genes (notably, many collagen isoforms) attributed to the claudin-low mesenchymal/stromal/fibroblast tumour subtype [49,53], but the importance of this classification in the context of cell lines awaits further investigation.

### MicroRNA expression profiling

**Figure 2:**
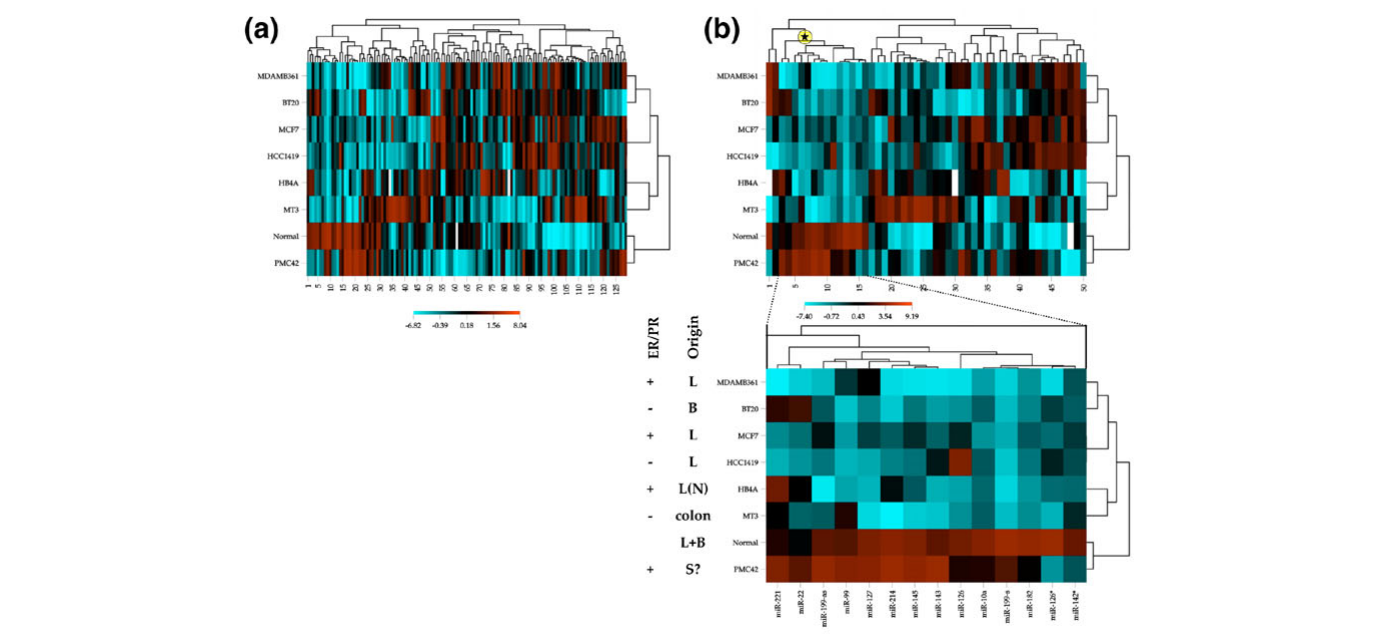
Analysis of microRNA (miRNA) gene expression in breast cancer cell lines and normal tissue. **(a) **Global miRNA heatmap. Log_2 _ratios of spots with intensity (A values) greater than 6 were used in CIMminer clustering analysis using correlation distances. **(b) **Heatmap of differentially expressed miRNAs. miRNA genes for which at least one value differed with log_2 _greater than 2.5 from the pool (either upregulated or downregulated) were used in CIMminer clustering analysis using correlation distance (upper). The PN miRNA cluster is indicated by a star and is enlarged (lower). The cell type origin (B, basal; L, luminal; N, normal; S?, possible stem cell) and the status of oestrogen receptor/progesterone receptor (ER/PR) are indicated on the left. PN miRNA, microRNA specifically expressed in PMC42 and Normal breast tissue.

Using an in-house array, we examined the expression of 171 miRNAs with a pooled cancer cell line RNA sample as reference. Clustering analysis of the 129 miRNAs with reliable readings was performed using several algorithms. A typical outcome is presented in Figure 2a. The main feature consistent with all algorithms used was the separation of normal breast tissue and PMC42 from all other cell lines. Importantly, the separation of PMC42 from other cell lines held true even when the normal sample was omitted from the analysis (data not shown), suggesting that its profile is inherently different and its separation is not driven by a minor similarity to the vastly different normal sample. On the other branch, the clustering did not partition according to tissue type (colon versus breast), progenitor type (normal versus cancer), ER/PR status, or luminal/basal nature of the cell, regardless of the algorithm employed. This was strikingly emphasised by MT3, an ER/PR-negative colon cancer cell line, sharing a branch with HB4a, an ER/PR-positive immortalised breast luminal epithelium line. It is also noteworthy that, although the mRNA expression of HCC1419 was closest to MDAMB361, its miRNA profile was most similar to that of MCF7, suggesting that the two profiles are not co-regulated.

Clustering analysis of the 50 differentially expressed miRNAs (defined in Materials and methods) showed two main branches spanning miRNAs that were either lost (16 miRNAs) or overexpressed (34 miRNA) in non-PMC42 cell lines compared with normal and PMC42 (Figure 2b, upper). The most striking cluster contained 14 miRNAs that were essentially only expressed in PMC42 and normal breast tissue (enlarged excerpt in Figure 2b). In contrast, miRNAs overexpressed in cell lines in comparison with normal/PMC42 seemed to be less consistent, only overexpressed in some of the non-PMC42 cell lines.

Examination of the miRNAs specifically expressed in PMC42 and Normal breast tissue (hereafter termed PN miRNA cluster) revealed many miRNAs repeatedly reported to be lost in tumours and cancer cell lines. These include miR-127 (for example, [55-57]), miR-199a sense and antisense (for example, [56-59]), and miRNAs 143 and 145 (for example, [56,58-62]). Importantly, PN miRNAs often show a correlated expression, most notably appearing as a near-continuous cluster differentiating between neoplastic and non-neoplastic samples in the prostate [56] and ovary [59]. Similarly, they are found to differentiate low-grade, normal-like, and luminal A breast tumours from the more aggressive breast cancer cases (high-grade, Her2, basal, and Luminal B) [63].

### Validation of expression of selected microRNA

**Figure 3:**
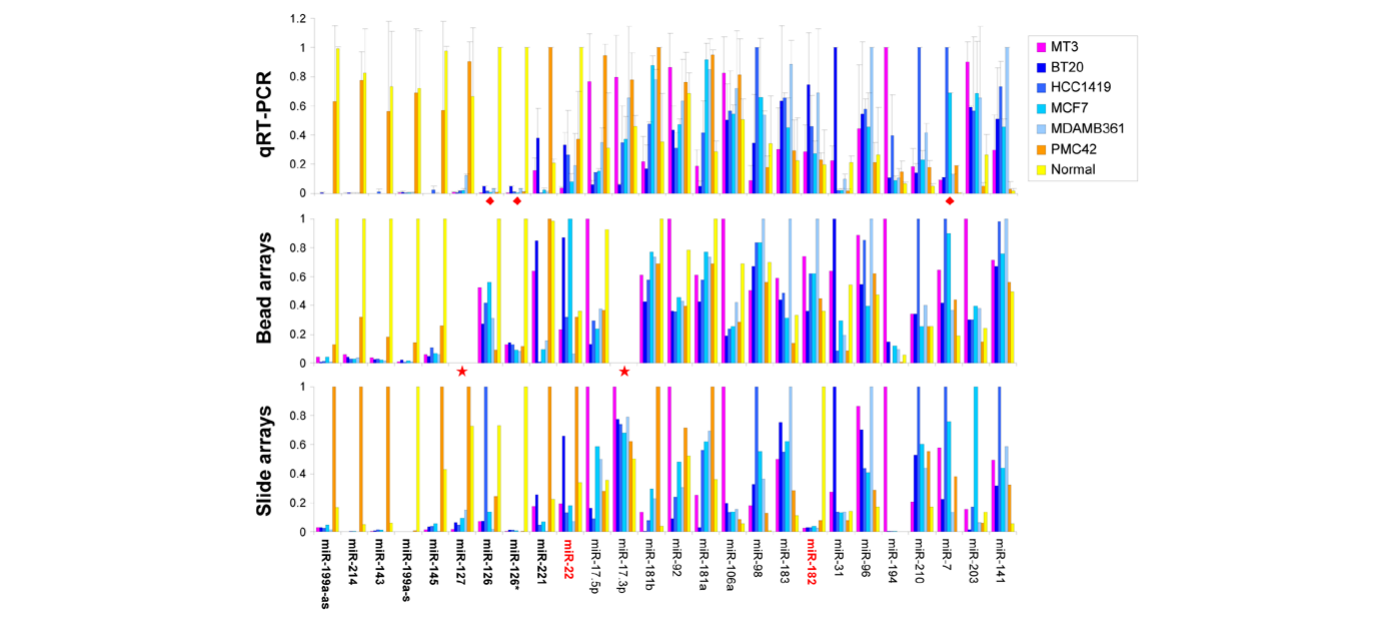
Validation of microRNA (miRNA) expression by Luminex bead arrays and real-time quantitative reverse transcription-polymerase chain reaction (qRT-PCR). The levels of 26 miRNAs measured using the methods indicated on the left are shown. qRT-PCR experiments were performed three times, each in triplicate, except for miRNAs 7, 126, and 126* (red diamond), which were measured once in triplicate; error bars indicate one standard deviation. On Luminex bead arrays, miRNAs 127 and 17-3p were below detection (red star). As units of expression in all methods are arbitrary, data were scaled to the highest-expressing sample for each miRNA. Normalised slide microarray data are presented on a non-logarithmic scale identical to the other two methods, for which the data were not normalised in any way. PN miRNAs appear in boldface. miRNAs with at least one negative value for correlation between the methods are shown in red. PN miRNA, microRNA specifically expressed in PMC42 and Normal breast tissue.

We validated the differential expression of the PN miRNAs by two independent methods for miRNA quantitation: single-channel Luminex bead arrays and real-time RT-PCR. Figure 3 summarises the results obtained from all three methods for 26 miRNAs, including 11 PN miRNAs as well as 15 non-PN control miRNAs with varying expression profiles. The alternative methods confirmed the specific loss of PN miRNAs, but not control miRNAs, from the non-PMC42 samples. The overall correlations between the methods were qRT-PCR/bead arrays 62%, qRT-PCR/slide arrays 65%, and bead arrays/slide arrays 43%. miRNAs 22 and 182 had at least one negative pair-wise correlation between the methods, and miR-126 appears to be expressed only in normal tissue, and these miRNAs were subsequently not considered as part of the PN cluster.

The limited correlation observed between the three platforms is the result of both conceptual and technical differences. For example, the initial choice of input RNA (total RNA, small-RNA fractions, and gel-purified mature miRNAs) is, in essence, a 'biological normalisation' step assuming that the rRNA or tRNA levels remain unchanged. We found inherent differences in the tRNA/rRNA ratio in different samples (7% to 20%), raising doubts as to the validity of either as a reference point. The extent of sample skewing during labelling by sequence-specific bias of RNA ligase [64] or PCR amplification is at present unknown. In addition, both array-based technologies necessitate a compromise in hybridisation conditions due to the range of miRNA melting temperatures. Most acutely, a low hybridisation temperature makes the distinction between miRNA family members (which differ by one to three nucleotides) very unreliable, thus presenting miRNA expression level readings that have contributions from several family members depending on their abundance and cross-reactivity in each assay. Further complexity in the interpretation of hybridisation signals arises from potential contributions of unprocessed precursor miRNAs where input samples have not been size-selected using PAGE. Lastly, as computational normalisation of miRNA expression data as yet awaits a golden standard, we analysed all our data in non-normalised mode or normalised to either 5S rRNA or the GEOmean of miRNAs 93 and 191, recently proposed as the most stable small-RNA reference [65]. Importantly, the differences in PN miRNA expression are specific and consistent, and the differences in PN miRNA levels are significantly larger than can be attributed to any of the aforementioned factors.

### Confirmation of PN microRNA loss in additional breast and lymphoblastoid cell lines

**Figure 4:**
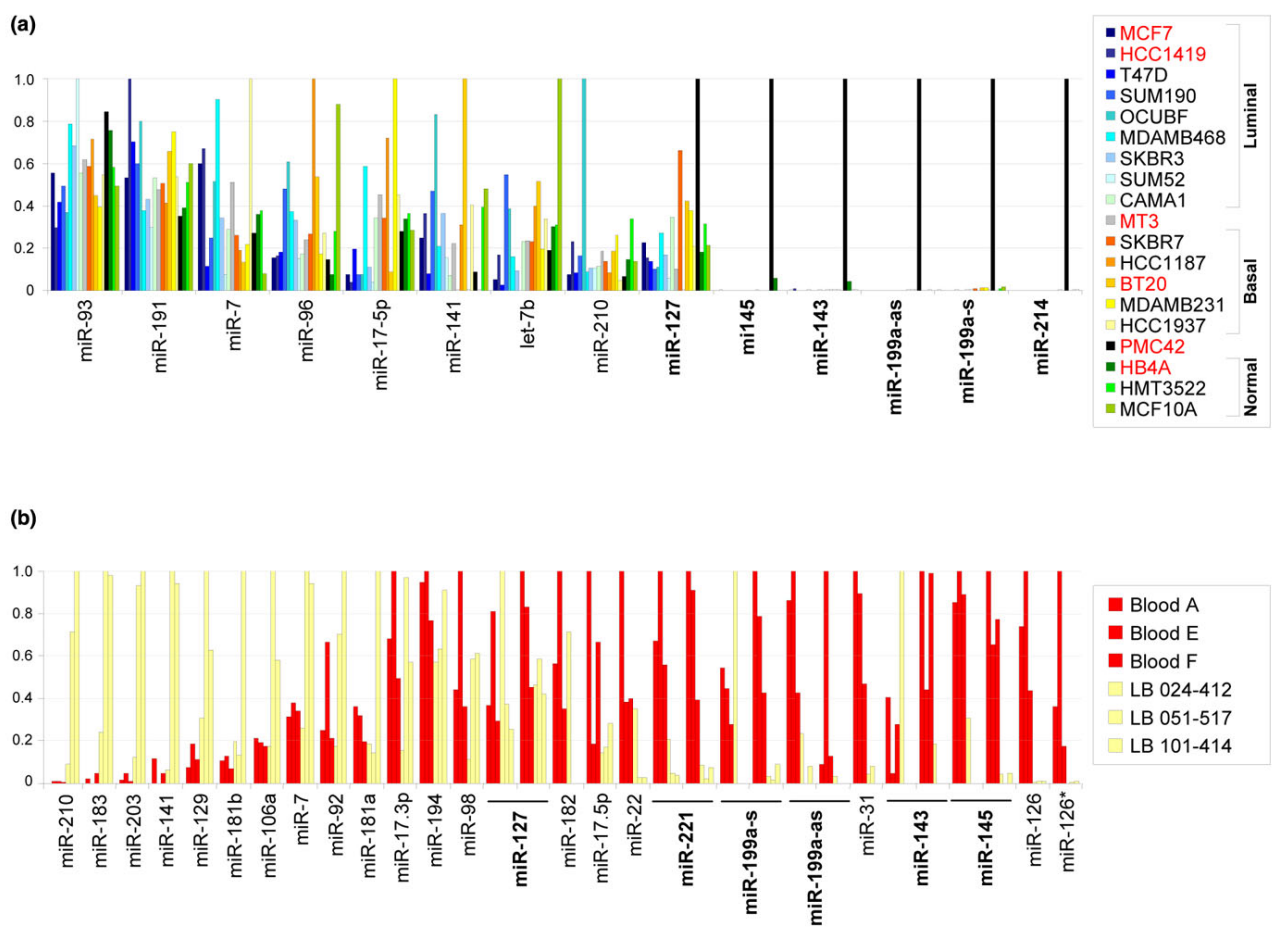
Real-time reverse transcription-polymerase chain reaction validation of PN miRNA loss in breast and lymphoblastoid (LB) cell lines. **(a) **Expression of the listed miRNA genes in 11 additional breast cancer and 2 immortalised normal cell lines alongside 6 of the initial panel of tested cell lines (red). The luminal/basal nature of each cell line is indicated on the right. Values were normalised to the GEOmean of miRNAs 93 and 191 [65]. Data were scaled to the highest-expressing sample for each miRNA. PN miRNAs appear in boldface. **(b) **The expression of the listed miRNA genes in B cells from 3 healthy blood donors and 3 LB cell lines. Values were normalised to the levels of 5S rRNA and scaled as above. The levels of miRNAs 127, 221, 199a-s, 199a-as, 143, and 145 were independently tested twice. PN miRNAs appear in boldface. miR-214 was below the detection threshold in all samples. PN miRNA, microRNA specifically expressed in PMC42 and Normal breast tissue.

To confirm the uniqueness of PMC42 in maintaining the expression of PN miRNAs, we examined the expression of selected PN and control miRNAs in 13 additional breast cell lines (2 immortalised normal epithelia and 11 from cancerous origin). Figure 4a summarises the obtained data, normalised to the GEOmean of miRNAs 93 and 191 [65]. The expression of miRNAs 214, 143, 145, and both 199a derivatives was completely lost in all examined cell lines except PMC42. The reduction in expression of miR-127 in the same samples was less prominent but still significant.

As the downregulation of many of the PN miRNAs has been reported in non-breast solid tumours and derived cell lines (such as ovary [59], pancreas [66], liver [58], colon [57,60,67,68], lung [62], and prostate [56]), we also examined the levels of the 26 miRNAs in CD19^+ ^B cells from three healthy blood donors and in three immortalised lymphoblastoid cell lines (Figure 4b). To ensure that no excessive RNA degradation occurred as a result of the inevitable delay in RNA extraction from normal bloods, small-RNA fractions were visualised following high-resolution electrophoresis, and no differences were discernible in the quantity, quality, or relative content of RNA derived from CD19^+ ^healthy B cells or lymphoblastoid cell lines (data not shown).

Apart from miR-127, which showed no difference between normal and immortalised lymphocytes, all validated PN miRNAs were downregulated in the lymphoblastoid cell lines, confirming the ubiquity of the PN expression profile. Loss of PN miRNAs in lymphoblastoid cell lines was specific as other miRNAs showed similar (or even increased) expression in the same samples. miR-214 was undetectable in any lymphocyte-derived samples, consistent with its reported low expression in lymph nodes and high expression in breast [69]. Conversely, the high levels of miR-126 measured in normal lymphocytes might account for its high expression in normal breast tissue despite its low levels in breast epithelial cell lines.

### Investigation of the mechanism of PN microRNA loss of expression

**Figure 5:**
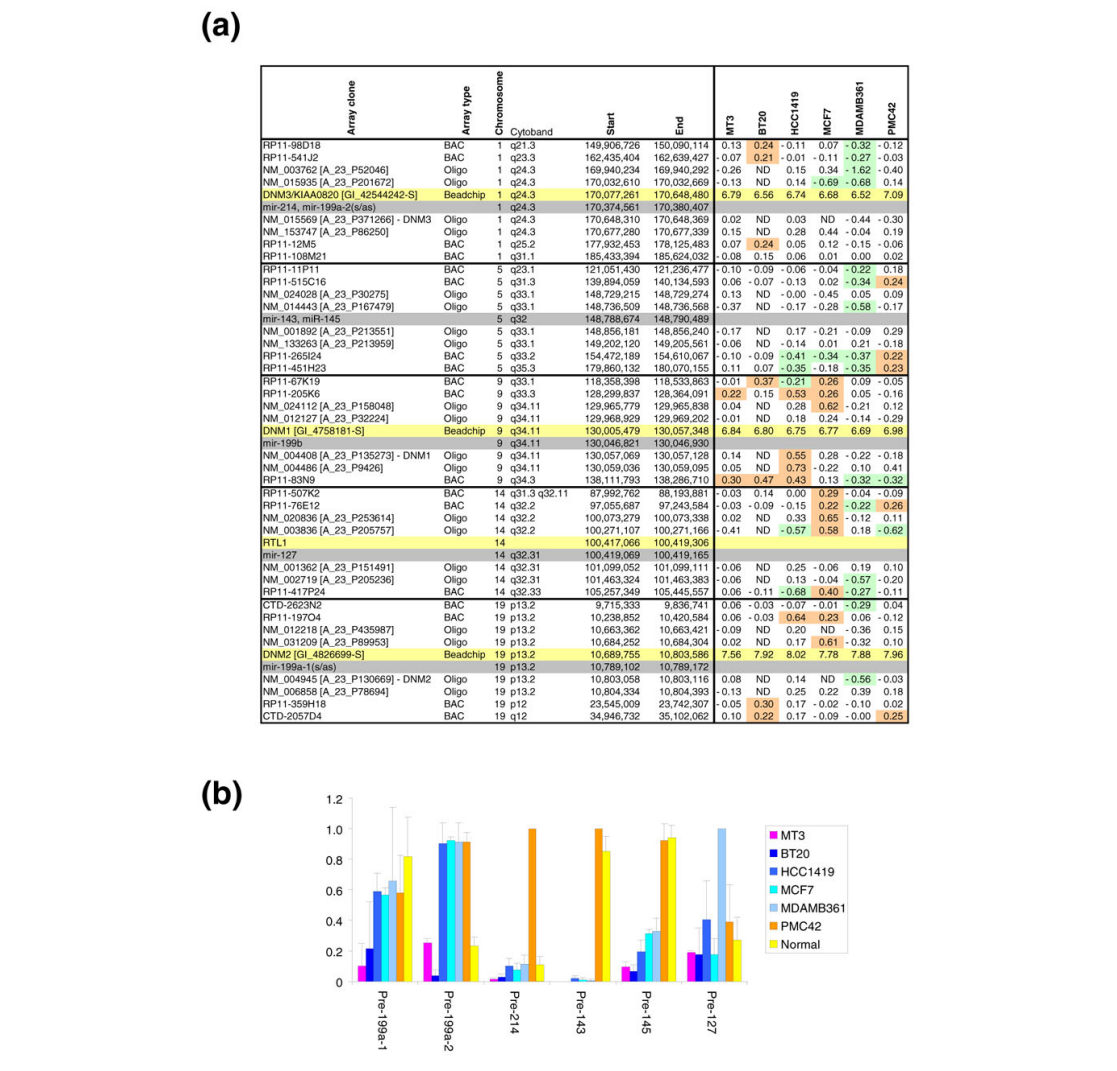
Mechanisms of PN miRNAs loss. **(a) **Examination of genomic copy number. Genomic copy numbers of the chromosomal regions surrounding candidate miRNAs from the PN cluster (grey highlight) were examined using bacterial artificial chromosome (BAC) and oligonucleotide array comparative genomic hybridisation and are listed by genomic location. Normalised log_2 _(copy numbers) of the two nearest clones on each array (names in square brackets) are reported unless unavailable due to the miRNA's proximity to the end of the chromosome. Gains are highlighted in orange; losses are highlighted in green. The mRNAs in whose introns the corresponding miRNAs reside are highlighted in yellow; where available, the log_2 _(mRNA expression) is given as measured by beadchip arrays. ND, not determined. **(b) **Real-time reverse transcription-polymerase chain reaction examination of pre-miRNA levels. As units of expression are arbitrary, data were scaled to the highest-expressing sample for each miRNA and were not otherwise normalised. Experiments were performed twice in triplicate; bars indicate one standard deviation. PN miRNA, microRNA specifically expressed in PMC42 and Normal breast tissue.

We then examined the potential mechanisms underlying the loss of PN miRNAs in non-PMC42 cell lines. For this analysis, we focused on the miRNAs that showed consistent results with the three independent methods (miRNAs of the 127, 143, 145, 199, and 214 families). As the karyotype of many cell lines is highly rearranged, we examined whether the loss of expression of the PN miRNAs in the five non-PMC42 cell lines can be attributed to losses of the corresponding genomic areas or parallel gains in PMC42. To this end, we analysed CGH (array CGH) data obtained from two types of arrays: oligonucleotide arrays (offering a better resolution), which were used to probe the copy number of chromosomal regions immediately adjacent to the miRNA of interest, and BAC arrays, which provided information about gene-poor genomic regions albeit with lower resolution (tiling BAC arrays were not available for our genomic areas of interest). The results of both are summarised in Figure 5a. Not only did we not find any consistent losses in areas adjoining PN miRNAs in non-PMC42 cell lines, some of them were in fact amplified in non-expressing cell lines (for example, chromosome 1 in BT20). Therefore, although some genomic losses (for example, chromosome 1 in MDAMB361) or breakpoints (for example, chromosome 14 in MCF7) may contribute to PN miRNA downregulation and specific micro-deletions spanning PN miRNAs cannot at present be eliminated, it seems unlikely that genomic copy number alterations are the main mechanism underlying changes in their expression.

We have also examined the changes in the expression of the mRNAs in whose introns some of the PN miRNAs reside. These can be considered as reporters for genomic presence and accessibility to transcriptional machinery (for instance, due to chromatin packing). miRNA/mRNA pairs included miR-199a-2 and miR-214 within DNM3 (opposite strands) and miR-199a-1 within DNM2 (opposite strand). miR-127 is located within RTL1 (retrotransposon-like-1) encoding a retrovirus-related polyprotein for which expression data were unavailable. We have also included miR-199b residing within DNM1 (opposite strands), which was omitted from the global miRNA analysis due to low A values but whose expression profile closely resembles that of miR-199a(s/as) (data not shown). Illumina beadchip data for the mRNAs listed above (Figure 5a, highlighted in yellow) show that not only is the corresponding genomic area not lost in either cell line, but they are either actively transcribed with similar efficiencies in all cell lines (basal levels of DNM3 consistent with its tissue-restricted expression; high expression of the ubiquitous DNM2; [70] and references therein) or even overexpressed in BT20 and MDAMB361 (EGFL7), suggesting that it is not chromatin accessibility that renders the PN miRNA genes inactive in non-PMC42 cell lines.

Last, using real-time RT-PCR, we examined the levels of the precursors of the PN miRNAs in the same samples so that the data in Figure 5b and Figure 3 (top panel) are directly comparable. The regulation of miRNAs 143 and 145 is at least partially achieved at the level of their precursors. In contrast, the precursor for miR-127 appears to be ubiquitously expressed in all cell lines and both precursors for miR-199a are present in many of the cell lines (though at lower levels in BT20) despite their corresponding mature products being preferentially formed or retained by normal and PMC42 cells only. Post-transcriptional mechanisms may be essential for the loss of miRNAs arising from multiple genomic loci, such as miR-199a-1 and miR-199a-2. Overall, it appears that the expression of the PN miRNAs is governed at more than one level with combinations of genomic, transcriptional, and post-transcriptional/processing mechanisms contributing to the loss of their mature form in non-PMC42 cell lines.

## Discussion

### PMC42 has normal-like mRNA and microRNA profiles

We show that PMC42, HB4a, and normal breast tissue have comparable mRNA profiles. The major feature segregating them from non-PMC42 cancer lines is high levels of mRNAs encoding hallmark components of the BM, such as collagen (various collagens, including type IV, preferred by mammary epithelium [4]), fibronectin (FN1), and laminins (subunits α1, α4, β1, and γ1). The latter are of great importance in the preservation of normal breast phenotype as modulators of both the proliferative response to stimulators like oestrogen [71] and of cell ability to induce β-casein [72]. ECM-related genes, and in particular laminins, were also reported to be perturbed in malignant breast epithelial cells compared with primary cultures of normal breast epithelium in a transcriptome analysis across several platforms [73]. PMC42 also exhibits markers of unregulated cell division emblematic of all cell lines. This was exemplified by its overexpression of 'breast cancer proliferation signatures', including BUB1, Polo-like kinase, and numerous cyclins, which are also upregulated in tumours compared with primary normal epithelium ([73] and references therein).

We also show that PMC42, but not any other cell line including several immortalised normal epithelial cell lines (HB4a, HMT3522, and MCF10A), possesses a normal-like miRNA profile. PMC42 is, to the best of our knowledge, the first breast cell line described to bear such a gene expression signature. We note the discrepancy between the normal-like mRNA and the heavily disrupted miRNA profiles of the immortalised normal epithelial lines, further underscoring the uniqueness of PMC42 as a model system. We focus on a group of miRNAs, whose increased expression in normal breast and PMC42 was ascertained using three independent methods and whose clustering is not algorithm-dependent. These PN miRNAs (miRNAs of the 127, 143, 145, 199, and 214 families) are almost ubiquitously associated with normal tissue, are absent in the brain [74], and are lost in the process of cell line establishment, as demonstrated here for breast and B cells and reported for many other tissues listed above. Interestingly, we were unable to detect miR-214 in B cells, suggesting that the expression, regulation, and possibly tropism of miR-214 is tissue-specific. Indeed, there are few reports of cancer-related loss of miR-214, and it has even been reported upregulated in lung tumours [62] alongside strongly downregulated miRNAs of the 143, 145, and 199 families. As it appears that tissue-specific signatures of miRNAs are of a combinatorial nature, miRNAs that are abundant in breast (199 and 214 families [69]) could potentially contribute to tissue-specific gene regulation as well as to more ubiquitous normal tissue homeostasis.

### Function and regulation of PN microRNAs

Although their native targets and phenotypic effects await detailed molecular investigation, some insights into the biological activity of PN miRNAs have been emerging recently. miRNA-214 has been assigned two roles: in HeLa cells it is required for apoptosis [75] while in zebrafish development it is involved in the specification of muscle cell fate [76], presumably representing one of its tissue-specific roles. In parallel, two independent papers demonstrated that the reintroduction of miRNAs 143 and 145 into cancer cell lines inhibits their growth [40,61], the latter also identifying ERK5 as a target of miR-143. A possible effector of miR-127 was identified as the proto-oncogene BCL6, a key player in B-cell apoptosis and neoplasia [55]. The reported pro-apoptotic and anti-proliferative activities of the PN miRNAs suggest that their loss in cell lines and tumours is not a coincidental or fortuitous observation but rather earmarks their role in normal cell fate. Moreover, it has been postulated that, rather than directly initiate developmental pathways, miRNAs canalise these events, confer robustness to otherwise-stochastic processes, and buffer genetic noise [77]. Presumably, their loss is imperative for concomitant perturbations in multiple molecular pathways typical of neoplastic transformation. The study of PN miRNAs so far has been significantly compromised by their loss from the cell line model systems best suited for their study, further underscoring the utility of PMC42 in future research.

Little is known about the regulation of the multi-step process leading to the formation of a mature miRNA including the genes' epigenetic modifications, transcriptional control, and the Drosha and Dicer processing of the resulting RNA. In our study, we have shown that no single mechanism accounts for the loss of the PN miRNAs in non-PMC42 cell lines. This finding is congruent with attempts to delineate the regulation of mature miRNA expression in other systems. At the epigenetic level, a combination of DNA demethylation and histone deacetylase inhibition can induce the expression of miR-127 in some, but not all, cancer cell lines without a parallel upregulation for other miRNAs at the same genomic location [55]. Analysis of several miRNA primary transcripts and their mature counterparts in a teratocarcinoma cell line and in mouse embryogenesis shows a discrepancy between the two expression profiles, indicating that many miRNAs are subject to post-transcriptional regulation [78]. However, similarly to our results, the levels of mature miRNAs 143 and 145 appeared to mirror those of their single primary transcript, suggesting that these miRNAs either uniquely escape regulation at the processing steps or are otherwise also fortuitously transcriptionally regulated in a similar way. Fittingly, correct processing of exogenous pre-miRNAs 143 and 145 in cell lines that do not express mature miR-143/145 was reported [60]. Interestingly, reduced mature, but not precursor, miR-143 and miR-145 in cancer cell lines have been previously noted, suggesting that their processing can also be regulated and highlighting the multilevel regulation of these miRNAs [67].

### A cell line model for normal breast epithelium

Studies of normal breast epithelium have been limited to primary cultures and handicapped by aspects of their purification and handling, such as finite life span and limiting quantity. Furthermore, each primary culture is unique, making any direct comparisons between data obtained by different laboratories difficult. Alternatively, immortalised normal cell lines can be used. However, concern was voiced about the little-studied effects of immortalisation on cellular functions either through direct effects (for example, the effect of HPV16-E6/E7 on p53 and retinoblastoma protein [79]) or through the extensive karyotype rearrangements visible in HB4a [24]. Indeed, the miRNA profile of HB4a is quite different from that of normal breast despite similarities in their mRNA profiles.

In this study, we show that the gene expression profile defining normal breast tissue is shared by PMC42. Despite the inherent limitations of all cell lines, PMC42 satisfies many of the features of a normal breast epithelium model. Its differentiation provides in a single sustainable culture both epithelial and myoepithelial cells supported by autonomous synthesis of BM components. The coexistence of near-normal repertoires of the regulatory miRNAs and their cognate targets is of great value to further studies of both mRNA and miRNA pathways, although further experiments are required to elucidate whether this coexistence is fortuitous or whether it mirrors the regulatory mechanisms governing the mRNA and miRNA profiles in normal breast epithelium.

Lastly, PN miRNAs potentially play a role in maintaining 'normal' cell proliferation or mortality. PMC42 was established from a presumed breast progenitor cell. It is tempting to postulate that stem or progenitor cells possess natural mechanisms that moderate the function of PN miRNAs and allow self-renewal and immortality in the absence of malignant transformation. Such cells would thus require fewer changes in the process of cell line establishment and could potentially maintain a more normal-like gene expression signature, including PN miRNAs. The confirmation of this hypothesis awaits further experiments.

## Conclusion

PMC42 is an unusual breast cancer cell line in its similarity to normal breast epithelium in cell composition and physiological responses. We present evidence that PMC42 also exhibits an mRNA and miRNA profile highly similar to the normal breast, including mRNAs encoding components of the BM and a group of miRNAs that are key candidates to have tumour-suppressor-like roles. Hence, PMC42 represents a unique biological tool to gain insight into the molecular events underlying epithelial/myoepithelial differentiation and the links between ECM, mRNA, and miRNA expression.

## Abbreviations

ATCC = American Type Culture Collection; BAC = bacterial artificial chromosome; BM = basement membrane; CGH = comparative genomic hybridisation; ECM = extracellular matrix; EGF = epithelial growth factor; ER = oestrogen receptor; miRNA = microRNA; PAP = poly(A) polymerase; PBS = phosphate-buffered saline; PCR = polymerase chain reaction; PN miRNA = microRNA specifically expressed in PMC42 and Normal breast tissue; PR = progesterone receptor; qRT-PCR = quantitative reverse transcription-polymerase chain reaction; RT = reverse transcription; SSC = Saline Sodium Citrate; TE = Tris + EDTA [ethylenediaminetetraacetic acid]; TMAC = tetramethylammonium chloride.

## Competing interests

The authors declare that they have no competing interests.

## Authors' contributions

AG and IS were responsible for experimental design, overall analysis, and all mRNA and miRNA experiments, excluding Luminex bead arrays, which were performed by CB. AG and IS contributed equally to this work. The miRNA slide microarrays were designed and printed by JS and FJL. MD participated in the analysis of mRNA expression. CGH data were contributed by JCMP (BAC arrays) and SFC and YW (execution and analysis of oligonucleotide arrays, respectively). CC conceived the study and participated in its design and coordination. The manuscript was drafted by AG and edited by IS and CC. All authors read and approved the final manuscript.

## Supplementary Material

Additional file 1 An Excel (Microsoft Corporation, Redmond, WA, USA) file detailing the gene ontology annotation of differentially expressed genes, accompanying Figure 1. Click here for file
